# Supplementary material for: Bayesian mediation analysis using patient-reported outcomes from AI chatbots to infer causal pathways in clinical trials
Source: PLoS One. 2025 Jul 24;20(7):e0326517. doi: 10.1371/journal.pone.0326517 (PMC12289003; doi:10.1371/journal.pone.0326517)
Supplement: Attachment — Submitted filename: PONE-D-25-04459 (1).pdf [file pone.0326517.s001.pdf]

PLOS ONE

Bayesian Mediation Analysis Using Patient Reported Outcome Collected from AI Chat to Estimate Causal Pathways in Clinical Trials

--Manuscript Draft--

**Manuscript Number:**	PONE-D-25-04459
**Article Type:**	Research Article
**Full Title:**	Bayesian Mediation Analysis Using Patient Reported Outcome Collected from AI Chat to Estimate Causal Pathways in Clinical Trials
**Short Title:**	Bayesian Mediation Analysis Using PRO from AI Chat Bot
**Corresponding Author:**	Shihao Shen<br>Agios Pharmaceuticals<br>Cambridge, UNITED STATES OF AMERICA
**Keywords:**	PRO; Causality; Bayesian; AI; chat bot; clinical trial
**Abstract:**	The integration of artificial intelligence (AI) chatbots into clinical trials offers a transformative approach to collecting patient-reported outcomes (PROs) by enabling real-time, interactive data collection. This study explores the use of AI chatbots to gather data on fatigue, stress, and adverse effects, leveraging Bayesian mediation analysis to uncover causal pathways linking treatment effects to outcomes via mediators such as adverse events and covariates. Using a simulation-based approach with GPT-4o, synthetic patient-chatbot dialogues were generated to validate the Bayesian mediation framework, which effectively decomposed total effects into direct and indirect components while quantifying uncertainty through credible intervals. The results demonstrated low bias, robust coverage, and reliable estimation of mediation pathways, underscoring the model's potential to enhance the granularity and accuracy of clinical trial data analysis. By integrating AI-driven PRO collection with Bayesian mediation methods, this study highlights a scalable, adaptive framework for refining causal inferences, improving patient engagement, and advancing personalized care in diverse healthcare settings.
**Order of Authors:**	Shihao Shen
	Jun Yin
**Additional Information:**	
**Question**	**Response**
**Financial Disclosure**<br><br>Enter a financial disclosure statement that describes the sources of funding for the work included in this submission. Review the submission guidelines for detailed requirements. View published research articles from *PLOS ONE* for specific examples.<br><br>This statement is required for submission and **will appear in the published article** if the submission is accepted. Please make sure it is accurate.	The author(s) received no specific funding for this work.

Powered by Editorial Manager® and ProduXion Manager® from Aries Systems Corporation

**Funded studies**<br>Enter a statement with the following details:<br>• Initials of the authors who received each award<br>• Grant numbers awarded to each author<br>• The full name of each funder<br>• URL of each funder website<br>• Did the sponsors or funders play any role in the study design, data collection and analysis, decision to publish, or preparation of the manuscript?<br><br>Did you receive funding for this work?	
**Competing Interests**<br><br>Use the instructions below to enter a competing interest statement for this submission. On behalf of all authors, disclose any competing interests that could be perceived to bias this work—acknowledging all financial support and any other relevant financial or non-financial competing interests.<br><br>This statement is required for submission and **will appear in the published article** if the submission is accepted. Please make sure it is accurate and that any funding sources listed in your Funding Information later in the submission form are also declared in your Financial Disclosure statement.<br><br>View published research articles from *PLOS ONE* for specific examples.	The authors have declared that no competing interests exist.

Powered by Editorial Manager® and ProduXion Manager® from Aries Systems Corporation

**NO authors have competing interests**<br><br>Enter: *The authors have declared that no competing interests exist.*<br><br>**Authors with competing interests**<br><br>Enter competing interest details beginning with this statement:<br><br>*I have read the journal's policy and the authors of this manuscript have the following competing interests: [insert competing interests here]*<br><br>* typeset	
**Ethics Statement**<br><br>Enter an ethics statement for this submission. This statement is required if the study involved:<br><br>• Human participants<br>• Human specimens or tissue<br>• Vertebrate animals or cephalopods<br>• Vertebrate embryos or tissues<br>• Field research<br><br>Write "N/A" if the submission does not require an ethics statement.<br><br>General guidance is provided below. Consult the submission guidelines for detailed instructions. **Make sure that all information entered here is included in the Methods section of the manuscript.**	N/A

Powered by Editorial Manager® and ProduXion Manager® from Aries Systems Corporation

**Format for specific study types**<br><br>**Human Subject Research (involving human participants and/or tissue)**<br>• Give the name of the institutional review board or ethics committee that approved the study<br>• Include the approval number and/or a statement indicating approval of this research<br>• Indicate the form of consent obtained (written/oral) or the reason that consent was not obtained (e.g. the data were analyzed anonymously)<br><br>**Animal Research (involving vertebrate animals, embryos or tissues)**<br>• Provide the name of the Institutional Animal Care and Use Committee (IACUC) or other relevant ethics board that reviewed the study protocol, and indicate whether they approved this research or granted a formal waiver of ethical approval<br>• Include an approval number if one was obtained<br>• If the study involved *non-human primates*, add *additional details* about animal welfare and steps taken to ameliorate suffering<br>• If anesthesia, euthanasia, or any kind of animal sacrifice is part of the study, include briefly which substances and/or methods were applied<br><br>**Field Research**<br><br>Include the following details if this study involves the collection of plant, animal, or other materials from a natural setting:<br>• Field permit number<br>• Name of the institution or relevant body that granted permission	
**Data Availability**<br><br>Authors are required to make all data underlying the findings described fully available, without restriction, and from the time of publication. PLOS allows rare exceptions to address legal and ethical concerns. See the PLOS Data Policy and FAQ for detailed information.	Yes - all data are fully available without restriction

A Data Availability Statement describing where the data can be found is required at submission. Your answers to this question constitute the Data Availability Statement and **will be published in the article**, if accepted.<br><br>**Important:** Stating 'data available on request from the author' is not sufficient. If your data are only available upon request, select 'No' for the first question and explain your exceptional situation in the text box.<br><br>Do the authors confirm that all data underlying the findings described in their manuscript are fully available without restriction?	
**Describe where the data may be found in full sentences. If you are copying our sample text, replace any instances of XXX with the appropriate details.**<br><br>• If the data are **held or will be held in a public repository**, include URLs, accession numbers or DOIs. If this information will only be available after acceptance, indicate this by ticking the box below. For example: *All XXX files are available from the XXX database (accession number(s) XXX, XXX.).*<br>• If the data are all contained **within the manuscript and/or Supporting Information files**, enter the following: *All relevant data are within the manuscript and its Supporting Information files.*<br>• If neither of these applies but you are able to provide **details of access elsewhere**, with or without limitations, please do so. For example:<br><br>*Data cannot be shared publicly because of [XXX]. Data are available from the XXX Institutional Data Access / Ethics Committee (contact via XXX) for researchers who meet the criteria for access to confidential data.*<br><br>*The data underlying the results presented in the study are available from (include the name of the third party*	All simulation data will be available from the Github.

and contact information or URL).<br>• This text is appropriate if the data are owned by a third party and authors do not have permission to share the data.<br><br>* typeset	
Additional data availability information:	Tick here if the URLs/accession numbers/DOIs will be available only after acceptance of the manuscript for publication so that we can ensure their inclusion before publication.

Powered by Editorial Manager® and ProduXion Manager® from Aries Systems Corporation

Manuscript

Click here to access/download;Manuscript;Bayesian Mediation Analysis Using PRO from AI Chat Bot.docx

Bayesian Mediation Analysis Using Patient Reported Outcome Collected from AI Chat to Estimate Causal Pathways in Clinical Trials

Shihao Shen[1*], Jun Yin[2]

[1]Agios Pharmaceuticals, Cambridge, MA, US, [2]Moffitt Cancer Center, Tampa, FL, US

* Corresponding author

E-mail: shihao.shen@agios.com

Abstraction

The integration of artificial intelligence (AI) chatbots into clinical trials offers a transformative approach to collecting patient-reported outcomes (PROs) by enabling real-time, interactive data collection. This study explores the use of AI chatbots to gather data on fatigue, stress, and adverse effects, leveraging Bayesian mediation analysis to uncover causal pathways linking treatment effects to outcomes via mediators such as adverse events and covariates. Using a simulation-based approach with GPT-4o, synthetic patient-chatbot dialogues were generated to validate the Bayesian mediation framework, which effectively decomposed total effects into direct and indirect components while quantifying uncertainty through credible intervals. The results demonstrated low bias, robust coverage, and reliable estimation of mediation pathways, underscoring the model's potential to enhance the granularity and accuracy of clinical trial data analysis. By integrating AI-driven PRO collection with Bayesian mediation methods, this study highlights a scalable, adaptive framework for refining causal inferences, improving patient engagement, and advancing personalized care in diverse healthcare settings.

Introduction

Artificial intelligence (AI) chatbots is being studied as a scalable and interactive method for collecting patient-reported outcomes (PROs) in clinical trials, providing real-time monitoring and personalized patient engagement. For instance, in an oncology setting in Brazil, an AI-based chatbot initiated the majority of patient dialogues, 97% of 3,883 conversations, facilitating early detection of adverse events and reinforcing adherence to treatment [1]. Also in diabetic retinopathy research, the PROBot framework harnesses large language models to capture enhanced PRO measures, helping improve compliance and reduce vision impairment [2]. Similarly, “Lucy LiverBot”, deployed in Australia for patients with decompensated cirrhosis, demonstrated not only an increase in health literacy but also unforeseen benefits for mental well-being [3]. These studies underscore the potential of AI chatbots to refine data collection, bolster patient engagement, and ultimately advance clinical trial outcomes.

In addition to improving data collection in clinical trials, AI chatbots confer several other benefits by facilitating real-time, interactive patient-reported outcomes (PROs) [4-6]. Their user-friendly format promotes consistent symptom and side-effect reporting, while built-in automation enables scalability across large patient populations and real-time responses to emerging health needs. These tools also enhance data accuracy by reducing human error, offer greater convenience and accessibility—especially for individuals with limited mobility or living in remote areas—and can significantly lower operational costs by minimizing manual data collection. Further, AI-driven analysis of patient data detects early warning signs and generates actionable insights, advancing personalized and patient-centered care by delivering targeted education and emotional support throughout treatment.

AI chatbots enhance insights into the causal pathways leading to patient-reported outcomes (PROs) by leveraging their capabilities in data collection, analysis, and contextual understanding [7]. By capturing both structured responses (e.g., symptom severity and timing) and unstructured data in the form of patient narratives or nuanced symptom descriptions, these tools build a rich, holistic dataset for uncovering cause-and-effect dynamics. Their real-time, longitudinal tracking of patient responses further enables the detection of evolving symptom patterns, while adaptive questioning drills deeper into potential contributing factors—such as lifestyle, environment, or psychological states—that traditional surveys might overlook. Advanced machine learning algorithms then integrate these multifaceted data streams, identifying subtle correlations and mind-body interactions (e.g., stress-related gastrointestinal symptoms) with predictive modeling that can inform personalized interventions. When deployed at scale, chatbots aggregate data across diverse populations, clarifying common causal pathways, subgroup-specific variations, and the relative effectiveness of different interventions. Altogether, these capabilities empower researchers and clinicians to generate a more complete and actionable understanding of the complex forces that drive health outcomes.

Bayesian mediation models play a pivotal role in elucidating the pathways through which an exposure exerts its influence on an outcome, especially when the goal is to separate total effects into direct and indirect components [8, 9]. Unlike their frequentist counterparts, these models incorporate prior information and generate posterior distributions for each parameter, enabling more nuanced inferences about mediator and exposure relationships. By quantifying uncertainty in a probabilistic framework, Bayesian methods allow researchers to better account for sampling variability and model complexity—particularly when dealing with small sample sizes, missing data, or intricate mediation structures. This approach provides a richer, more flexible platform for integrating new and existing evidence, thus enhancing the precision and interpretability of causal inferences in clinical trials and other complex healthcare studies.

Here, we leverage patient-reported outcomes gathered through an AI chatbot to fit a Bayesian mediation model aimed at uncovering the causal pathways linking the exposure to the outcome. By incorporating both structured and unstructured data from chatbot interactions—such as symptom severity, behavioral changes, and nuanced patient narratives—into our modeling, we gain a more granular view of how indirect effects operate through mediators. This Bayesian approach allows us to integrate prior information and continuously update our inferences as more data becomes available, a key advantage when investigating complex clinical scenarios. Moreover, the probabilistic framework of Bayesian

mediation enables a more transparent quantification of uncertainty, particularly beneficial when analyzing large-scale, real-world data from AI-driven PRO collection in clinical trials.

Materials and Methods

We design an AI chatbot to gather the PRO of fatigue, stress and adverse effect, using the following router in Figure 1. The chatbot begins by asking how the user feels, then specifically inquires whether the user has experienced fatigue. If the answer is "Yes," it explores possible social or cultural reasons for the fatigue before delving into whether treatment side effects might be causing it. On the other hand, if the user reports "No" fatigue, the chatbot asks about social or cultural stress and subsequently checks for any side effects of the treatment. This branching logic tailors follow-up questions based on the user's initial response, guiding the conversation toward identifying potential psychological, social, or medical factors related to the user's condition.

Figure 1. Router of the chatbot to gather the PRO of fatigue, stress and adverse effect.

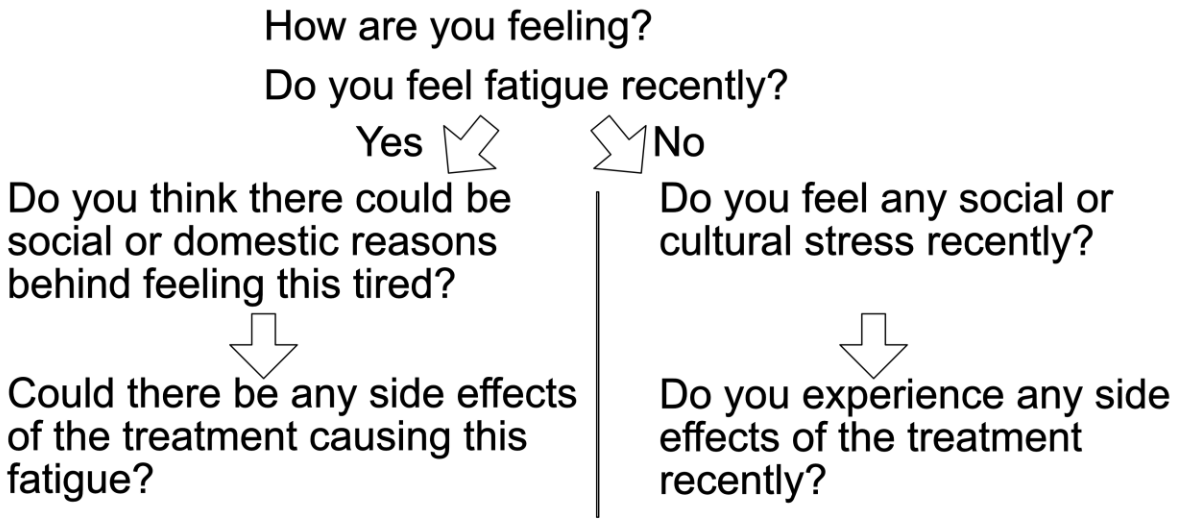

We conducted a simulation of user–chatbot interactions using GPT-4o [10], serving as both the "agent" (the AI chatbot) and the "user" (the patient). First, we defined a series of potential health-related queries and responses reflecting real-world patient concerns, such as fatigue, cultural or social stress, and treatment side effects. Then, we implemented a scripted branching logic—mirroring the conversation flow depicted in our decision-tree diagram—to guide the generation of GPT-4 responses at each user input. Specifically, we employed role-based prompting in which GPT-4 was instructed to assume the "agent" role, formulating questions according to predefined conversational steps, and then switched to the "user" role to supply plausible patient statements or acknowledgments. Through iterative refinement, we ensured that the simulated dialogues accurately reflected the complexity and variability of genuine patient–chatbot interactions, including follow-up questions and context-sensitive inquiries. This approach generated a robust, synthetic dataset of conversation transcripts, which was later used to test and validate

our Bayesian mediation model, particularly in understanding how different types of user feedback (e.g., admitting fatigue vs. not) might shape causal pathways in patient-reported outcomes.

Using a Bayesian mediation framework in Figure 2, we capture the direct and indirect effects of X (treatment effect) on Y (fatigue) via M (adverse event), while also accounting for Z (stress) as a covariate. Conceptually, X influences M (path a), which in turn affects Y (path b), and X may also exert a residual direct effect on Y (path c'). Additionally, Z enters the model as a predictor of Y (path d). In practice, we place prior distributions (often weakly informative normal priors) on each path coefficient and use Markov chain Monte Carlo (MCMC) sampling in PyMC [11] to estimate the joint posterior distributions of the model parameters. This allows us to derive credible intervals for the indirect effect (i.e., the product $a \times b$) and the direct effect (c') while propagating uncertainty throughout. The result is a posterior-based estimate of whether and how the treatment (X) influences fatigue (Y) both directly and through the mediator (M), taking into account the role of stress (Z).

Using definitions from Hayes (2018) [12], the effect of an exposure (X) on an outcome (Y) can be decomposed into three key components: the direct effect (c'), capturing the influence of X on Y independent of the mediator (M); the indirect effect ($a{\cdot}b$), reflecting how X affects Y through the mediator; and the total effect ($c = c' + a{\cdot}b$), which is the sum of both paths. In a Bayesian mediation framework, each path coefficient is assigned a prior, and posterior distributions of c', $a{\cdot}b$, and c are derived via Markov chain Monte Carlo (MCMC) sampling. The posterior estimates, along with their credible intervals, allow researchers to ascertain not only whether mediation is present, but also the relative magnitudes of direct and indirect pathways, thereby providing a nuanced and probabilistic view of how X influences Y through M.

Figure 2. Bayesian mediation framework.

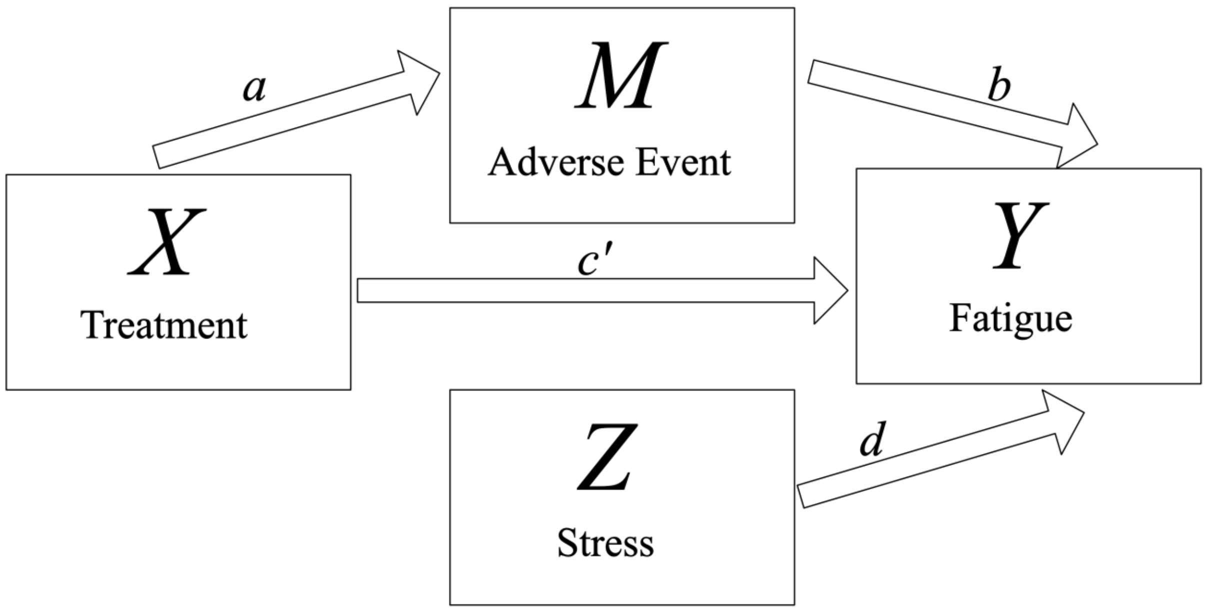

Results

We conducted 100 iterative simulations of chatbot–user dialogues using GPT-4o. In each simulation, 75 subjects were randomized 1:1 into active or placebo groups, defining their X variables. From each subject's dialogue, we extracted the values for M, Y, and Z, based on the simulated responses. Figure 3 provides an example of a single simulated chat, featuring a subject who reports fatigue, domestic stress, and an adverse event.

Figure 3. An example of the simulated chat

Chatbot: Hi there, how are you feeling today? Have you been experiencing any fatigue recently?

Subject: Hello. Yes, I've actually been feeling pretty exhausted for the past few days.

Chatbot: I see. Do you think there could be social or domestic reasons that might be contributing to how tired you feel?

Subject: Possibly. I've been juggling a lot at home—keeping up with my kids' activities, plus managing some financial stress. It's been overwhelming.

Chatbot: That sounds challenging. Do you think there could also be any side effects from your current treatment or medication that might be causing or adding to this fatigue?

Subject: It's something I'm starting to wonder about. It feels like I've had worse insomnia than usual.

Chatbot: Understood. It might be a good idea to speak with your healthcare provider about these concerns. They can help rule out or confirm if the new treatment is contributing to your fatigue and explore possible solutions.

Subject: Definitely—I'll schedule an appointment and see if there's anything we can adjust.

Chatbot: That's a great plan. Meanwhile, make sure to take care of yourself, both mentally and physically. If you feel your stress levels rising, try to reach out for support or consider simple stress-relief activities.

Subject: Thank you. I really appreciate the advice. It helps to know there are steps I can take to figure this out.

In our simulation result (Table 1), **a** represents the effect of the exposure (*X*) on the mediator (*M*), while **b** captures how changes in *M* translate into changes in the outcome (*Y*). The parameter **c′** (often referred to as the "direct effect") measures the influence of *X* on *Y* that is not channeled through *M*. We include **d** as a coefficient for an additional covariate—such as stress—that can also predict *Y*. Meanwhile, $\mathbf{i_m}$ and $\mathbf{i_y}$ are intercepts for the mediator and outcome equations, respectively, indicating baseline levels of *M* and *Y*.

The parameters σ_m and σ_y represent the residual (or unexplained) variability in *M* and *Y*, respectively. Finally, the indirect effect (a × b) quantifies the portion of *X*'s influence on *Y* transmitted through *M*, whereas the total effect is the sum of the indirect effect and the direct effect (*c′*), providing an overall measure of *X*'s impact on *Y*.

In our simulation outputs, **Bias** represents the average deviation of the estimated parameter from its true value, indicating whether the estimates tend to systematically over- or underestimate the parameter. **RMSE** (root mean square error) reflects both the variance of the estimator and its bias, offering a single metric for the overall accuracy of the estimate. **Coverage** denotes the proportion of simulated datasets for which the 95% credible interval (CI) includes the true value, thus capturing how well our interval estimates align with the real parameter. **Interval Width** is the mean width of those CIs, indicating how precise (narrow) or imprecise (wide) the intervals are.

Our simulation results indicate generally low bias and acceptable root mean square error (RMSE) for the primary path coefficients (*a*, *b*, *c′*, and *d*), with coverage values approaching or exceeding the conventional 0.90–0.95 range. For instance, *a* and *c′* exhibit minimal bias (0.00397 and 0.00303, respectively), robust coverage (0.97), and relatively narrow interval widths. Meanwhile, *b* shows a larger RMSE (0.248) and somewhat lower coverage (0.91), suggesting greater uncertainty in that parameter. Notably, the indirect effect has perfect coverage (1.00) and a small bias (0.00214), reflecting reliable estimation of the mediation pathway, although the total effect coverage (0.86) falls just below the typical target for credible intervals. Overall, these findings support the stability and accuracy of the Bayesian mediation model, while highlighting areas where parameter estimates may benefit from increased sample size, more precise priors, or further model refinement.

Table1. MCMC simulation results.

	Bias	RMSE	Coverage	Interval Width
a	0.00397	0.027764	0.97	0.13721
b	0.0264	0.248215	0.91	0.81048
**c′**	0.00303	0.105357	0.97	0.42059
d	-0.00146	0.113848	0.94	0.42598
i_m	-0.03691	0.04929	0.87	0.10089
i_y	0.06435	0.248244	0.87	0.81146
σ_m	-0.00444	0.041313	0.5	0.04341
σ_y	0.00832	0.029047	0.95	0.10416
indirect effect	0.00214	0.009217	1	0.04909
total effect	-0.11428	0.184343	0.86	0.59791

Discussion

This study highlights the transformative potential of integrating artificial intelligence (AI) chatbots into clinical trials for collecting patient-reported outcomes (PROs) and advancing causal inference through Bayesian mediation analysis. By leveraging AI chatbots, we were able to gather real-time, interactive data on fatigue, stress, and adverse effects while ensuring scalability and accessibility. The use of GPT-4 in

simulating patient-chatbot dialogues enabled the generation of a robust synthetic dataset, which provided a valuable framework for testing and validating Bayesian mediation models.

The findings demonstrated that Bayesian mediation analysis is a powerful tool for disentangling direct and indirect effects in causal pathways, particularly in complex clinical scenarios. The low bias, robust coverage, and reliable estimation of key parameters (e.g., indirect effects) observed in this study underscore the robustness of the Bayesian approach. Notably, the model's ability to incorporate prior information and quantify uncertainty through credible intervals makes it especially suitable for analyzing real-world data where complexities such as missing data and small sample sizes are common.

However, the results also identified areas for improvement. The parameter b, representing the effect of the mediator on the outcome, exhibited a larger root mean square error (RMSE) and slightly lower coverage, suggesting potential variability in the indirect pathway estimates. Additionally, the total effect coverage was marginally below the conventional threshold, indicating that refining model specifications or increasing the sample size may further improve estimation accuracy. Future work could explore the impact of more precise priors, larger datasets, or alternative modeling approaches to address these limitations.

The incorporation of AI chatbots in clinical trials not only streamlines PRO data collection but also enhances the granularity and contextual richness of the data. By capturing both structured responses and unstructured patient narratives, chatbots provide a comprehensive dataset for identifying nuanced cause-and-effect relationships. Furthermore, their adaptability and real-time functionality offer significant advantages in monitoring patient symptoms, detecting early warning signs, and delivering personalized interventions.

This study underscores the importance of combining AI-driven data collection with advanced analytical techniques like Bayesian mediation analysis to maximize the value of clinical trial data. The approach presented here has broad implications for improving patient engagement, refining causal inferences, and advancing personalized healthcare. As the adoption of AI chatbots continues to grow, future research should explore their integration into diverse clinical contexts and evaluate their long-term impact on patient outcomes and healthcare efficiency.

References

1. Murad Junior, M., et al., *The use of a chatbot based on artificial intelligence to collect patients reported outcomes in oncology practice. A pilot study with real-world data*, 2020, American Society of Clinical Oncology.
2. Pielka, M., et al. *[Vision Paper] PRObot: Enhancing Patient-Reported Outcome Measures for Diabetic Retinopathy using Chatbots and Generative AI*. in *2024 IEEE International Conference on Big Data (BigData)*. 2024. IEEE.
3. Au, J., et al., *A beta-prototype chatbot for increasing health literacy of patients with decompensated cirrhosis: usability study.* JMIR Human Factors, 2023. **10**(1): p. e42506.
4. Chen, Z., et al., *Chat-ePRO: Development and pilot study of an electronic patient-reported outcomes system based on ChatGPT.* Journal of Biomedical Informatics, 2024. **154**: p. 104651.

5. Soares, B., et al., *MT35 Feasibility of an AI-Based Interactive Voice Response (IVR) System for Patient-Reported Outcome Measures (PROMs) Collection: A Scalable Solution to Collect Patient-Centered Real-World Evidence.* Value in Health, 2024. **27**(12): p. S492.
6. Kaywan, P., et al., *Early detection of depression using a conversational AI bot: A non-clinical trial.* Plos one, 2023. **18**(2): p. e0279743.
7. Laymouna, M., et al., *Roles, users, benefits, and limitations of chatbots in health care: rapid review.* Journal of medical Internet research, 2024. **26**: p. e56930.
8. Enders, C.K., A.J. Fairchild, and D.P. MacKinnon, *A Bayesian approach for estimating mediation effects with missing data.* Multivariate Behavioral Research, 2013. **48**(3): p. 340-369.
9. Yuan, Y. and D.P. MacKinnon, *Bayesian mediation analysis.* Psychological methods, 2009. **14**(4): p. 301.
10. Achiam, J., et al., *Gpt-4 technical report.* arXiv preprint arXiv:2303.08774, 2023.
11. Abril-Pla, O., et al., *PyMC: a modern, and comprehensive probabilistic programming framework in Python.* PeerJ Computer Science, 2023. **9**: p. e1516.
12. Hayes, A.F., *Partial, conditional, and moderated moderated mediation: Quantification, inference, and interpretation.* Communication monographs, 2018. **85**(1): p. 4-40.
